# Increased Land Use by Chukchi Sea Polar Bears in Relation to Changing Sea Ice Conditions

**DOI:** 10.1371/journal.pone.0142213

**Published:** 2015-11-18

**Authors:** Karyn D. Rode, Ryan R. Wilson, Eric V. Regehr, Michelle St. Martin, David C. Douglas, Jay Olson

**Affiliations:** 1 U.S. Geological Survey, Alaska Science Center, 4210 University Drive, Anchorage, Alaska, 99508, United States of America; 2 United States Fish and Wildlife Service, Marine Mammals Management, 1011 E Tudor Road, Anchorage, Alaska, 99503, United States of America; 3 U.S. Geological Survey, Alaska Science Center, 250 Egan Drive, Juneau, Alaska, 99801, United States of America; 4 Brigham Young University, Plant and Wildlife Sciences, 5049 LSB, Provo, Utah, 84602, United States of America; Sonoma State University, UNITED STATES

## Abstract

Recent observations suggest that polar bears (*Ursus maritimus*) are increasingly using land habitats in some parts of their range, where they have minimal access to their preferred prey, likely in response to loss of their sea ice habitat associated with climatic warming. We used location data from female polar bears fit with satellite radio collars to compare land use patterns in the Chukchi Sea between two periods (1986–1995 and 2008–2013) when substantial summer sea-ice loss occurred. In both time periods, polar bears predominantly occupied sea-ice, although land was used during the summer sea-ice retreat and during the winter for maternal denning. However, the proportion of bears on land for > 7 days between August and October increased between the two periods from 20.0% to 38.9%, and the average duration on land increased by 30 days. The majority of bears that used land in the summer and for denning came to Wrangel and Herald Islands (Russia), highlighting the importance of these northernmost land habitats to Chukchi Sea polar bears. Where bears summered and denned, and how long they spent there, was related to the timing and duration of sea ice retreat. Our results are consistent with other studies supporting increased land use as a common response of polar bears to sea-ice loss. Implications of increased land use for Chukchi Sea polar bears are unclear, because a recent study observed no change in body condition or reproductive indices between the two periods considered here. This result suggests that the ecology of this region may provide a degree of resilience to sea ice loss. However, projections of continued sea ice loss suggest that polar bears in the Chukchi Sea and other parts of the Arctic may increasingly use land habitats in the future, which has the potential to increase nutritional stress and human-polar bear interactions.

## Introduction

The Arctic is experiencing rapid and extensive sea ice loss that is projected to continue as a result of increasing levels of greenhouse gas emissions [1]. Among Arctic marine mammals, the response of polar bears (*Ursus maritimus*) to ecological change has received the most attention relative to global warming impacts [2]. Much of the information about polar bears and sea ice loss, however, is based on only a few of the 19 recognized subpopulations [3], particularly the Western Hudson Bay, Southern Beaufort Sea, and Barents Sea subpopulations. Western Hudson Bay is seasonally ice free and the entire subpopulation comes onshore during the summer where they survive primarily by metabolizing accumulated stores of fat and lean mass [4]. In the southern Beaufort and Barents seas, the ice retreats northward in the summer. Some bears in both subpopulations come onshore but the majority appear to move north with the ice [5, 6]. With recent sea ice loss, bears in these three subpopulations have increasingly spent time on shore in the summer [5, 7, 8, 9]. However, both annual sea ice dynamics and polar bear life history strategies vary across the Arctic, and in many regions, baseline data on land use and its relationship with changing sea ice conditions are unknown.

The degree to which increased land use is a common response of polar bears to sea ice loss is relevant to the state of Arctic ecosystems, humans living and working in the Arctic, and polar bear conservation and management. Several studies in areas where bears are coming onshore earlier and spending more time on shore have documented localized but intense nest predation of sea birds and geese [9, 10, 11, 12, 13]. On Wrangel Island, Russia, polar bear predation resulted in the death of at least 226 walruses (*Odobenus rosmarus*) between 1989 and 2006, of which 81% died as a result of stampedes caused by polar bears [14]. Grizzly bears (*Ursus arctos*) occupy terrestrial habitats in many parts of the polar bear’s range, such that increased land use could increase sympatry and potential competition [15]. Hybridization between grizzly bears and polar bears has been documented in northwestern Canada [16]. Further, increases in terrestrial habitat use can lead to increased interactions with humans that may result in safety concerns for humans or in disturbance or death for polar bears [17].

In addition to the effects listed above, increased land use is likely to have nutritional consequences for polar bears. While on land, polar bears have little or no access to ice seals, their primary prey. Although numerous studies have documented that polar bears consume terrestrially-derived foods [18], scavenge beached marine mammal carcasses, eat remains of Native subsistence harvests [5], and actively hunt hauled-out walruses [14], most terrestrial feeding is opportunistic and of lower energetic content compared to the marine prey that polar bears consume on the sea ice [19, 20]. Thus, terrestrial foods are likely capable of supporting only a small proportion of current polar bear numbers. In seasonally ice-free habitats of eastern Canada, such as western Hudson Bay, analyses of polar bear blood chemistry [21], feeding habits via stable isotopes [22, 23] and body mass and fat changes indicate that polar bears are primarily fasting while on land [4]. Longer periods fasting on shore has been suggested as the primary mechanism by which body condition, reproduction, and ultimately polar bear survival in Western Hudson Bay have declined in conjunction with sea ice loss [7, 24, 25].

A large portion of the polar bear’s circumpolar range occurs in areas where sea ice retreats towards the Arctic basin and away from land masses during the summer [26], requiring bears to make decisions as to whether to stay on land or remain with the retreating ice both during the summer and for denning. Little is known about the feeding patterns of polar bears that remain on the sea ice over the Arctic basin throughout the summer. Prey densities in deep waters beyond the continental shelf are thought to be low [27], and polar bears that summer on the sea ice in the southern Beaufort Sea appear to have energy expenditures typical of fasting, non-hibernating mammals [28]. Although a majority of bears in the southern Beaufort Sea remain with the sea ice during the summer, pregnant females primarily den on land [29]. In areas such as this where land-based denning has increased [29] or changed in distribution [30], the ability of bears to maintain access to terrestrial denning areas without compromising foraging opportunities before or after denning may also influence cub survival [31]. There is some evidence that important spring foraging habitats near terrestrial denning areas in Svalbard have declined [32]. Thus, the decisions a bear makes as to whether to use land prior to and during maternal denning may have nutritional implications for pregnant females [33].

Because increased land use by polar bears has implications for Arctic ecosystem ecology, bear-human interactions, and polar bear health, here, we examined land use by adult female polar bears in the Chukchi Sea; members of a subpopulation which range between Alaska and Russia. We compared land use during two time periods (1986–1995 and 2008–2013), when substantial reductions in sea ice habitat occurred [34]. A portion of bears in this subpopulation have typically summered [14, 35] and denned on Wrangel and Herald Islands [36] and the northeast coast of Chukotka [37]. Sea-ice dynamics are similar to the adjacent southern Beaufort Sea, as well as regions throughout Russia and Norway, where the sea ice retreats towards the pole during the annual sea ice minimum [26]. Thus, we sought to determine if the Chukchi Sea subpopulation has similarly increased duration on land during the summer and increased use of terrestrial habitats for denning, which would further support that increased land use may be a common response of polar bears to sea ice loss. Our objectives were to 1) determine which land masses were used by female polar bears; 2) compare the proportion of female polar bears in the Chukchi Sea subpopulation using land, and the amount of time spent on land, between the two time periods; and 3) evaluate environmental factors that influence the location, timing, and duration of land use.

## Materials and Methods

### Ethics Statement

This research was approved under the Marine Mammal Protection Act and Endangered Species Act with U.S. Fish and Wildlife Service (USFWS) permit number MA046081. Capture protocols were approved by the U.S. Geological Survey, (USGS) Alaska Science Center and USFWS Region 7 Institutional Animal Care and Use Committees.

### Polar bear capture and collaring

**Fig 1:**
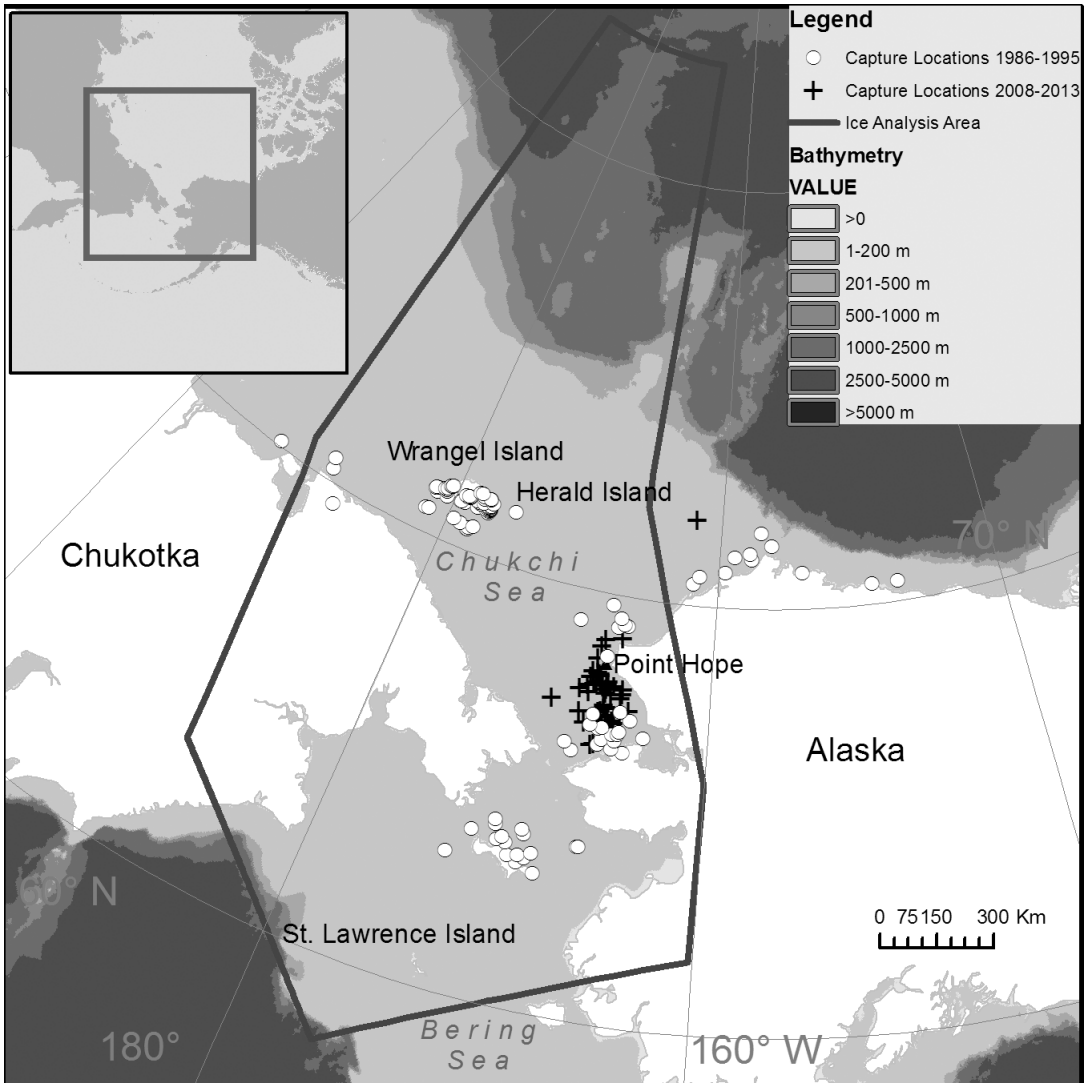
Locations where adult female polar bears were captured and fitted with satellite radio collars in 1986–1995 and 2008–2013. The area used for ice analysis is outlined in black.

Polar bears were captured and released on sea ice or land between mid-March and early May in the Chukchi and northern Bering Seas 1986–1995 and 2008–2013 (Fig 1). The study area ranged from 58 to 83°N and -160 to 166°W. In 1986–1995, 74 capture events occurred on or near Wrangel Island or Herald Island, 34 on sea ice off the Alaskan mainland coast, 13 on sea ice off of St. Lawrence Island, Alaska, and 4 on sea ice off the northeast Chukotkan coast. In 2008–2013, all polar bear captures occurred on sea ice off the Alaskan mainland coast (Fig 1). Polar bears were located from a helicopter and immobilized with a dart containing either M-99 in 1986 or zolazepam-tiletamine (Telazol^®^ or Zoletil^®^) [38] in 1986–2013.

Only adult female bears (≥ 4 years old) can safely be fitted with collars because they are no longer growing. Adult males were not collared because their necks are larger than their heads, preventing collar retention. Between 1986 and 1995, 103 adult females were fitted with satellite radio collars (Telonics Inc., Mesa, AZ), some of which were captured multiple times and/or wore collars for multiple years resulting in 127 bear-years of data. Collars deployed between 1986 and 1995 transmitted to the Argos System (Argos, www.argos-system.org) every 3 days. Between 2008 and 2013, 47 adult females were fitted with satellite radio collars providing 62 bear-years of data. Argos-only collar deployments in 2008–2010 provided Argos locations every one to three days; Global Positioning System (GPS) collar deployments 2010–2013 provided GPS locations four times per day. A bear-year was defined from the time of capture in the spring through at least the following January, so that summer habitat use and denning behavior could be identified.

### Estimating land use from location data

Prior to 2010, all radio collar locations were determined by the Argos System with accuracy from < 250 m to > 1500 m (see http://www.argos-system.org/web/en/78-faq.php#faq-theme-55). From 2010 to 2013, GPS locations were transmitted via Argos. Location data were filtered to remove implausible locations using the Douglas Argos-Filter algorithm [39], which retained all standard quality class locations (classes 3, 2, and 1), rejected all class Z locations, and retained auxiliary class locations (0, A, and B) if they were corroborated by a consecutive location within 10 km, or if movement rates were < 10 km hr^−1^ and turning angles were not extremely acute. Instances of dropped collars were identified based on activity and temperature sensors, and data collected post-drop were removed.

To integrate tracking data obtained from GPS and Argos, we employed the continuous time correlated random walk (CRAWL) model [40] to predict locations on a regularized, daily time interval (daily). The CRAWL model accounts for variable location quality and sampling intervals. For Argos locations, we used accuracy estimates provided by Telonics, Inc. (i.e., 3: 150 m, 2: 350 m, 1: 1000 m, 0: 1500 m; http://www.telonics.com/technotes/argosintro.php). Because location accuracies were not provided by Telonics for auxiliary location classes A or B, we prescribed conservative location accuracies of 5,000 and 10,000 m, respectively. We assigned locations obtained from GPS collars an accuracy of 30 m [41].

Using the observed locations and associated accuracies, we used the R statistical computing [42] package ‘CRAWL’ [43] to predict daily polar bear locations from 1 Jul to at least 31 Jan the following year, a period that encompasses the annual sea ice minimum and the initiation of denning. Because the uncertainty of CRAWL location estimates increases as the time between observed locations increases [44], we excluded predicted locations that occurred between observed locations separated by >14 days. The majority of data gaps filled with the CRAWL model were less than 5 days apart (1986–1995: 7.2% of locations were >4 days apart; 2008–2013: 1.3% of locations were > 4 days part). To validate that the CRAWL model was able to predict land use when there was a 14 day gap between observations, we found all observed land use locations (i.e., midpoints in a 14 day series of locations) where there was another observed location 7 days before and 7 days after that location. We then removed all points in the 14 day period, and used CRAWL to predict whether the midpoint occurred on land. We found that the majority of predicted locations occurred on land (mean = 0.74, SE = 0.03), which validated our inclusion of location data with up to 14-day gaps.

We also performed cross-validation by resampling observed data, to evaluate what proportion of predicted locations would be misclassified as occurring on land or ice. For each individual’s path, we withheld five observed locations and their associated land status (on land or on ice). We then predicted the location of the animal at the same time as the withheld location and determined whether the predicted point occurred on land or ice. For data collected 1986–1995, 4.4% of withheld locations were misclassified, whereas the percent of misclassified locations for data collected 2008–2013 was 2.1%. Thus, the CRAWL model performed well at correctly predicting the land-use status of withheld locations.

We considered a bear to be on land if its predicted location was within 5 km of land as identified by the Global Self-consistent, Hierarchical, High-resolution, Geographic Database (GSHHG version 2.3.4; http://www.soest.hawaii.edu/pwessel/gshhg/). The 5 km buffer was used to encompass small barrier islands that may receive heavy use by polar bears in the summer [5] but were not depicted as land in the GSHHG, and to account for low accuracy of some locations. Our 5 km buffer might have resulted in some offshore bears being classified as on land, and vice versa, but this was less likely to occur during the focal time period of our analysis (August through October) because landfast ice is largely absent during this period [45] and the pack ice (the nearly continuous mass of ice that retreats northward during the summer) has generally receded >>5km north of the coast. Bears within 5 km of the coast during this time were likely to have been on land. Because ARGOS does not transmit when a bear is in the water, it is unlikely that locations received within 5 km on shore occurred in water.

In the case of denning, we used a control chart-based algorithm [46] that classified denning events based exclusively on collar temperature. We defined denning as a consecutive bout of higher than expected temperatures (determined using temperatures from non-denning bears) lasting 35 days or more during winter months (October-May). This control chart-based algorithm for identifying denning behavior agreed with 94.5% of denning events confirmed via observations during VHF radio tracking (n = 73)[46] and 96.3% of den identifications based on temperature, activity, and location data in Fischbach et al. [30]. Because the physical features of dens in snow banks often attenuate transmission, location data were typically unavailable or of low quality during the denning period. If at least one observed location at the start of, during, or at the end of the denning period occurred on land, it was assumed that the den occurred on land. Den events identified could have been successful maternity dens, unsuccessful maternity dens that were abandoned early, or shelter dens used during periods of poor weather or reduced food availability [47].

We compared the proportion of bears that spent more than 7 days onshore and more than 21 days onshore (which included the bears that spent > 7 days onshore) between August and October using a binary logistic regression (i.e., 0 = did not use land > 7 or > 21 days, 1 = used land > 7 or > 21 days). We used the threshold of 21 days to indicate longer-term summering behavior that has been documented elsewhere [5] and 7 days as an indicator of shorter-term land use. Shorter-term land use was included because little is known about the duration of land use by Chukchi Sea polar bears. We also used binary logistic regression to compare substrate use (i.e., land versus ice habitats) during denning between the two time periods of our study.

The number of days bears spent onshore (for bears spending > 7 or > 21 days onshore) between August and October was compared using an ANOVA because no individual bear was repeated in the data set (i.e., although some bears provided location data for more than one year, no individual provided data indicating land use > 7 or > 21 days for more than one year). Due to the potential for differences in capture location between time periods to affect the number of bears coming onshore, where they came to shore for denning and summering, and how long they spent there, we also compared these metrics using only bears that were captured in the same portion of the Alaska coast in 1986–1995 as where captured in 2008–2013. To understand the degree to which bears may use land during times of the year other than summer, we also used an ANOVA to compare the percent of locations among entire bear-years that were spent on land for bears with a minimum of 300 days of location data post-deployment. For linear models and logistic regressions, β-values are reported with standard errors (SE). For ANOVA comparisons, means are reported with standard deviations (stdev).

### Environmental factors: Ice metrics

We were interested in the influence of summer sea ice conditions on where bears come to shore, how long they spend on shore, and whether they denned on land or sea ice. The sea ice minimum in the Arctic and in the Chukchi Sea occurs in September. Therefore, we focused on ice conditions during the months on either side of September (i.e., Aug-Oct) using daily sea ice concentration data from the National Snow and Ice Data Center (accessed 16 Oct 2014) 1986–2013 [48].

Several studies suggest that bears will remain on sea ice until the concentration drops below 15–30% [8, 49] and that they prefer ice habitats over shallow, continental shelf waters <300 m deep [8, 49, 50]. We hypothesized that the availability of ice between August and October would determine how long bears spent onshore. We determined the average daily proportion of the continental shelf in the ice analysis area (outlined in black in Fig 1) that was covered by ice of ≥15% concentration between August and October [34], and used this in a regression analysis with annual average days spent onshore as the response variable.

We also hypothesized that sea ice conditions may affect where bears summer and den on land among the multiple land masses available within the Chukchi Sea, including the northeast Chukotkan coast, northwest Alaskan coast, and Wrangel and Herald Islands. To determine whether ice conditions were related to where bears came ashore in summer and for land-based denning (i.e., the response variable), we estimated the dates that sea ice (of ≥15% concentration) retreated from (hereafter “ice retreat date”) and returned to (hereafter “ice return date”) within <50 km from 25 x 25 km pixels of rasterized coastal segments along the Chukotkan coast, Alaskan coast, and Wrangel and Herald Islands. From the daily grids of sea ice concentration, we created grids of the average weekly sea ice concentration. We then used the R [42] package ‘SDMTools’ [51] to identify the largest, contiguous, area of weekly average ice with ≥ 15% concentration likely to be indicative of the mass of pack ice that retreats northward in the summer. If pack ice never retreated >50 km from a coastal segment, we assigned retreat and return date of 365 and 1, respectively. We chose 50 km from shore to determine ice retreat and return dates because it corresponds to a “long distance swim” for polar bears [52], which we hypothesized would correspond to an ecologically significant distance for individual animals.

To determine if either ice retreat or return dates were related to where polar bears used land, either for denning or during the summer for >7 days, we used conditional logistic regression [53] to relate used areas to areas where use was not observed. For this analysis, we included all bears that were observed > 7 days onshore between August and October regardless of whether they had data gaps during that period. We used the ‘coxph’ function in the survival package [54] for R [42] to determine the potential relationship between ice formation and retreat dates and areas of summering and denning in a given year (i.e., in logistic regression 0 = unused and 1 = used). We built separate models for denning locations and summer land use locations. The time series of annual ice retreat dates was highly correlated with ice return dates (*r* = -0.80). Therefore, these variables were examined independently in models to determine which variable was most explanatory based on Akaike Information Criterion (AIC) scores (S1 Table)[55]. Each model included ice retreat or ice return date and an interaction term between time period (1986–1995 and 2008–2013) and the ice variable. The model with sea ice retreat was more explanatory than the model with ice return date (S1 Table), thus we used this model in our analysis. Because of the simplicity of the models, we did not implement a model selection process but rather assessed whether the model coefficients were significant at *P* ≤ 0.05. To ensure that there were no biases in denning and summer land use locations (northwest Alaskan coast, Wrangel/Herald Islands, northeast Chukotkan coast) based on where a bear was captured (i.e., northwestern Alaskan coast, St. Lawrence Island, Wrangel Island, northeast Chukotkan coast), we performed contingency table analyses [56] comparing capture location to ultimate denning or summering location.

For examinations of the relationship between ice retreat and return dates and locations of dens on land, we included 6 land-based maternal den locations that were identified by direct observation rather than satellite tracking.

## Results

### Summer land use

The percent of collared female bears using land > 7 days between August and October increased from 20.0% in 1986–1995 (16 of 80 bear-years) to 38.9% in 2008–2013 (14 of 36 bear-years; *ß* = 0.9 ± 0.4 SE, *P* = 0.03) when including all bears sampled in both time periods. This increase was also apparent when including only bears captured off the Alaska coast 1986–1995 (13.0%; 3 of 23 bear-years)(*ß* = 1.4 ± 0.7, *P* = 0.05). The percent of bears on land for > 21 days between August and October increased from 12.5% in 1986–1995 (10 of 80 bear-years) to 36.1% in 2008–2013 (13 of 36 bear-years; *ß* = 1.4 ± 0.5, *P* = 0.005). None of the bears captured off the Alaska coast 1986–1995 stayed onshore > 21 days resulting in a similar increase in land use when using this subsample of bears (*ß* = 2.4 ± 1.1, *P* = 0.02). Results from the sample of all bears captured during both time periods include both bears that denned (n = 28) and did not den (n = 87) during the coming winter, and exclude bears for which data gaps (e.g., gaps in observed locations > 14 days as described in the methods) prevented determination of habitat use during the summer (n = 73). Temperature data available for one bear that summered on land was insufficient to determine whether or not she denned. A similar percentage of the earlier (35.0%) and latter (38.1%) sample were denning bears. The majority of bears (76.7%; 23 of 30) that spent > 7 days on shore spent > 21 day onshore.

**Fig 2:**
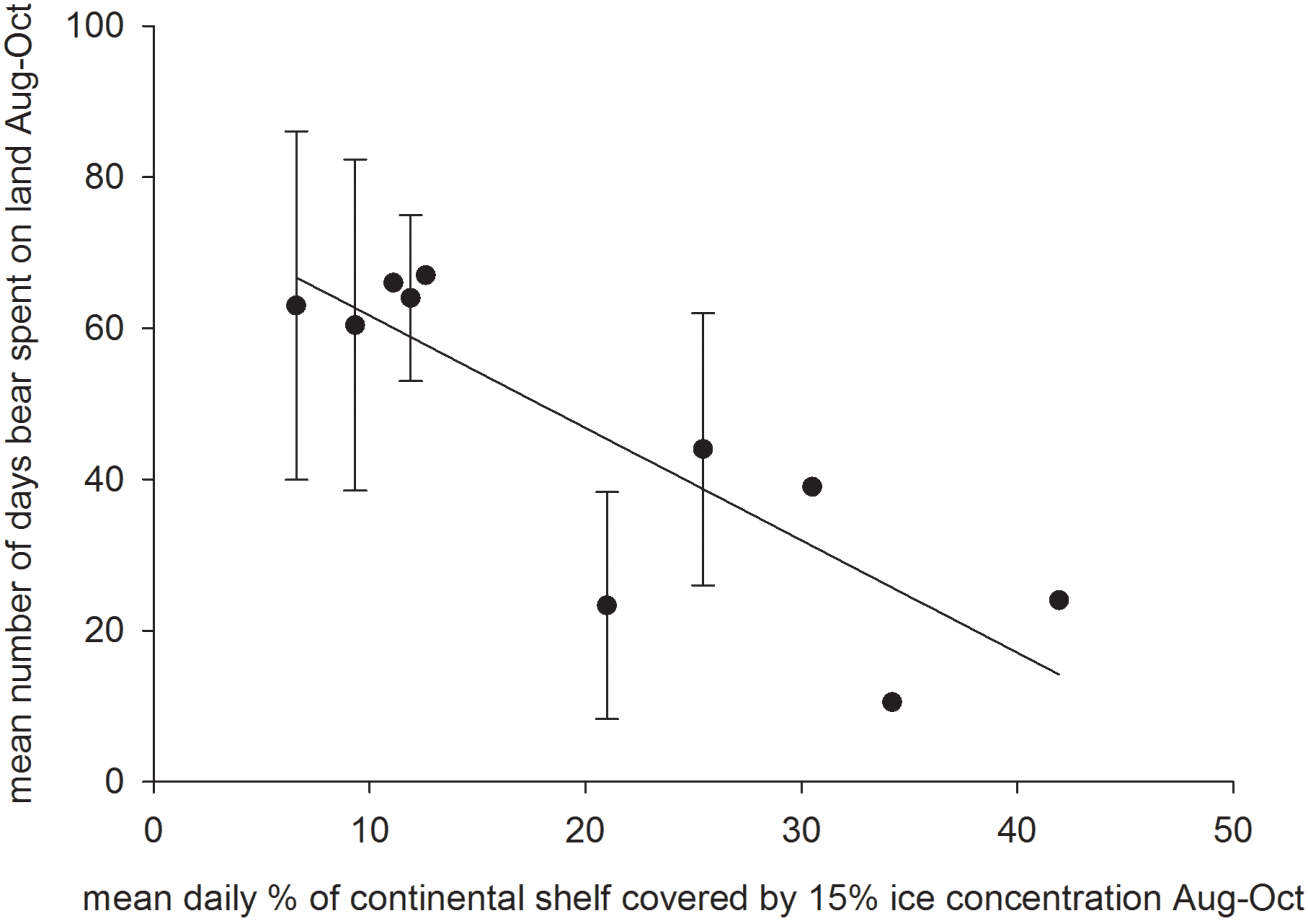
Relationship between the mean daily percent of the continental shelf covered by sea ice (≥15% concentration) between the months of August and October and mean (± 1 stdev) number of days denning and non-denning female polar bears were on land during those months for each of 10 years in which data were available for at least two individuals (1987, 1988, 1990–1993; 2009–2011, 2013). Only bears that spent more than 7 days on land were included. Sample sizes are provided in parentheses above data points.

Land use increased in duration by 30.0 ± 7.6 (SE) days for collared bears that spent > 7 days total (i.e., not consecutive) onshore between August and October including denning and non-denning bears (1986–1995: 32.7 ± 21.1 days; mean ± stdev, *n* = 16; 2008–2013: 62.6 ± 20.2 days, *n* = 14, *F*_*1*,*28*_ = 15.7, *P* < 0.0001). Similarly, land use increased in duration by 21.2 ± 7.2 days for bears that spent > 21 days onshore (1986–1995: 44.7 ± 17.5 days, *n* = 10; 2008–2013: 65.9 ± 16.7 days, *n* = 13, *F*_*1*,*21*_ = 8.8, *P =* 0.007). Land use increased in duration when including only bears captured off the Alaska coast 1986–1995 (14.7 ± 4.0 days, n = 3; *F*_*1*,*15*_ = 16.0, *P =* 0.001). Whether a bear came onshore only for the summer or to subsequently den, did not affect the timing of their arrival onshore for bears that spent > 7 days onshore (*F*_*1*,*28*_ = 0.2, *n* = 30, *P* = 0.6). Denning and non-denning bears came onshore 20.2 ± 7.9 days earlier in 2008–2013 (Julian date: 241.2 ± 20.2, *n* = 14) than in 1986–1995 (Julian date: 261.4 ± 22.7, *n* = 16, *F*_*1*,*28*_ = 6.5, *P* = 0.016). Of 14 polar bears that came onshore between August and October and later denned on land, four (2 of 6 in 1986–1995 and 2 of 8 in 2008–2013) left land prior to denning and then returned to land to den. Bears that came onshore between August and October and did not den or did not stay onshore into the denning period departed land 22.3 ± 7.6 days later in 2008–2013 (Julian date: 309.7 ± 6.7, *n* = 7) than 1986–1995 (287.4 ± 19.3, *n* = 12, *F*_*1*,*17*_ = 8.6, *P* = 0.009). There was no difference in the mean date when denning bears left land post-denning (1986–1995 Julian date = 98.5 ± 15.8, *n* = 13; 2008–2013 = 87.8 ± 26.7, *n* = 13, *F*_*1*,*24*_ = 1.5, *P* = 0.2). The mean duration of time denning and non-denning bears spent on land during a given year, when including bears that spent > 7 days on land, from August to October was related to the mean area covered by ≥15% ice concentration August through October in the ice analysis area (*R* ^*2*^ = 0.73, *P* = 0.002; Fig 2). This relationship was also apparent when using the individual bear data, rather than means across all bears for a given year (*R* ^*2*^ = 0.42, *P* = 0.0001). The average daily proportion of the continental shelf within the study area covered by ≥15% ice concentration between August and October decreased from 29.4% (± 9.1) in 1986–1995 to 11.1% (± 3.7) in 2008–2013 (*F*_*1*,*14*_ = 21.6, *P* < 0.0001).

Considering location data throughout entire bear-years rather than just the summer months (i.e., bear-years with a minimum of 300 days of location data), 53.3% and 45.7% of individual bear-years had less than 5% their locations on land during 1986–1995 and 2008–2013, respectively (binary logistic regression 0 = > 5% locations on land, 1 = < 5% locations on land; *ß* = - 0.31 ± 0.41, *P* = 0.46). On average, individual bear-years in 2008–2013 included a greater percentage of locations on land (22.9 ± 25.7%, *n* = 35) than in 1986–1995 (11.1 ± 15.6%, *n* = 75; *β* = -11.8 ± 4.0, *F*_*1*,*108*_ = 8.9, *P* = 0.004).

### Summer and denning locations

In 1986–1995 and 2008–2013, 69.2% and 89.5% of polar bears that spent > 7 days on land between August and October came to Wrangel Island, respectively. Fifteen percent of bears that spent > 7 days on land between August and October came to Chukotka in 1986–1995, and no bears came to Chukotka during these months in 2008–2013. The 4 bears that summered on Chukotka between 1986 and 1995 included one caught off the Alaska coast, one caught of St. Lawrence Island, and two caught on Wrangel Island. Less than 10% of bears spent > 7 days on the Alaskan coast (7.7% in 1986–1995; n = 26 and 5.3% in 2008–2013; n = 19) or Herald Island (7.7% in 1986–1995 and 5.3% in 2008–2013) between August and October. Wrangel and Herald Islands were commonly used for summering by bears captured on Wrangel Island (80%; 12 of 15 bears) and off the Alaska coast (67%; 4 of 6 bears) suggesting that capture location may not strongly influence summering location. Fewer bears summered on the northwest Alaskan and northeast Chukotkan coasts including 6.6% and 13.3%, respectively, of those captured on Wrangel Island and 16.7% and 16.7% respectively, of those captured off the northwest coast of Alaska. Differences in summering relative to capture location may be a result of the small sample of bears (n = 6) that were captured off the northwest coast of Alaska in 1986–1995 that also summered on land.

**Fig 3:**
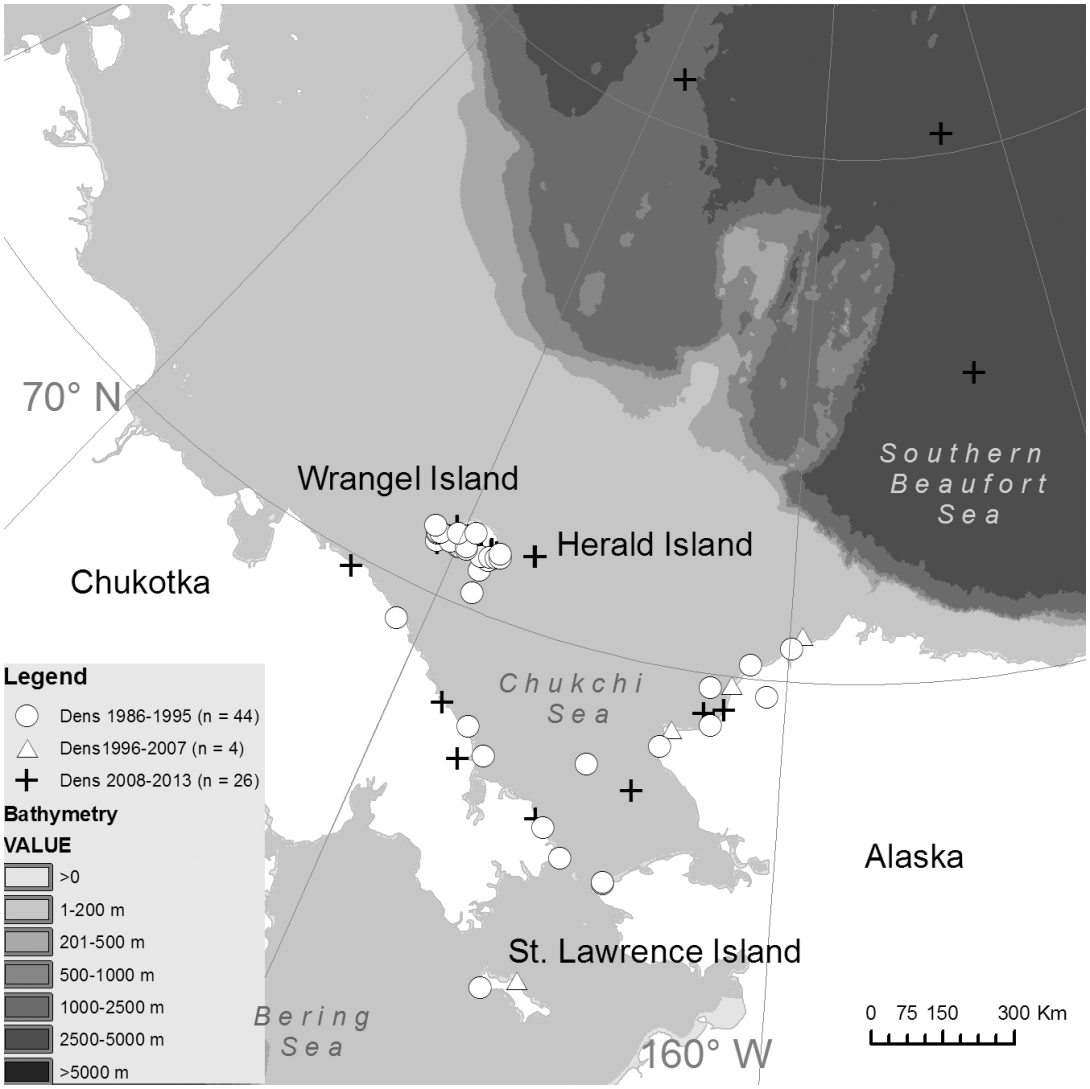
Locations of female polar bear winter dens 1986–1995, 1995–2007 and 2008–2013. The majority of den locations were determined from satellite telemetry locations of collared bears. Six den locations on land were determined from observation. Dens are assumed to be maternity denning attempts although some bears may have exited early without cubs.

A total of 70 denning events were documented; 64 from temperature data of collared bears and 6 from direct observation (the latter of which were excluded in comparisons of land versus ice-based denning). Forty-four were documented between 1986 and 1995, four between 1996 and 2007, and 26 between 2008 and 2013 (Fig 3). Most dens during both time periods occurred on land (1986–1995: 92.3%; 2008–2013: 84.0%) and substrate use (i.e., land or ice) did not differ between time periods (binary logistic regression: *ß* = 0.22, *P* = 0.70; Table 1). Denning location patterns were similar when including only bears caught in the same area for both time periods (e.g., off the northwest coast of Alaska) with 87.8% of dens occurring on land between 1986–1995 compared to 92.3% when using the entire sample of bears. Similarly, the proportion of land-based dens occurring on Wrangel Island was similar when including only bears captured off the Alaska coast (i.e. same capture locations as the 2008–2013 sample) in 1986–1995 with 55.5% occurring on Wrangel Island compared to 56.1% when using the entire sample of bears.

**Table 1:** Percent of Chukchi Sea female polar bears denning on or within 5 km of land in different geographic areas during two time periods based on observed locations from collared bears (n = 56) and directly observed dens (n = 6). Denning location could not be determined for all bears identified to have denned based on collar temperature data. North (N) Alaska included land in Alaska north of Point Hope and south (S) Alaska included land in Alaska south of Point Hope.

	N Alaska	S Alaska	Chukotka	Herald Island	Wrangel Island
1986–1995 (n = 41)	17.1	7.3	14.6	4.9	56.1
2008–2013 (n = 21)	9.5	0	19.0	9.5	61.9

All bears spent > 60 days at the den site. Of those re-observed the following spring, none had yearlings—which would most conclusively support shelter denning rather than maternity denning (i.e., whereas observations of a lone bear or bear with cubs of the year post-denning could be maternity or shelter denning).

### Relationships between land use location and sea ice conditions

**Fig 4:**
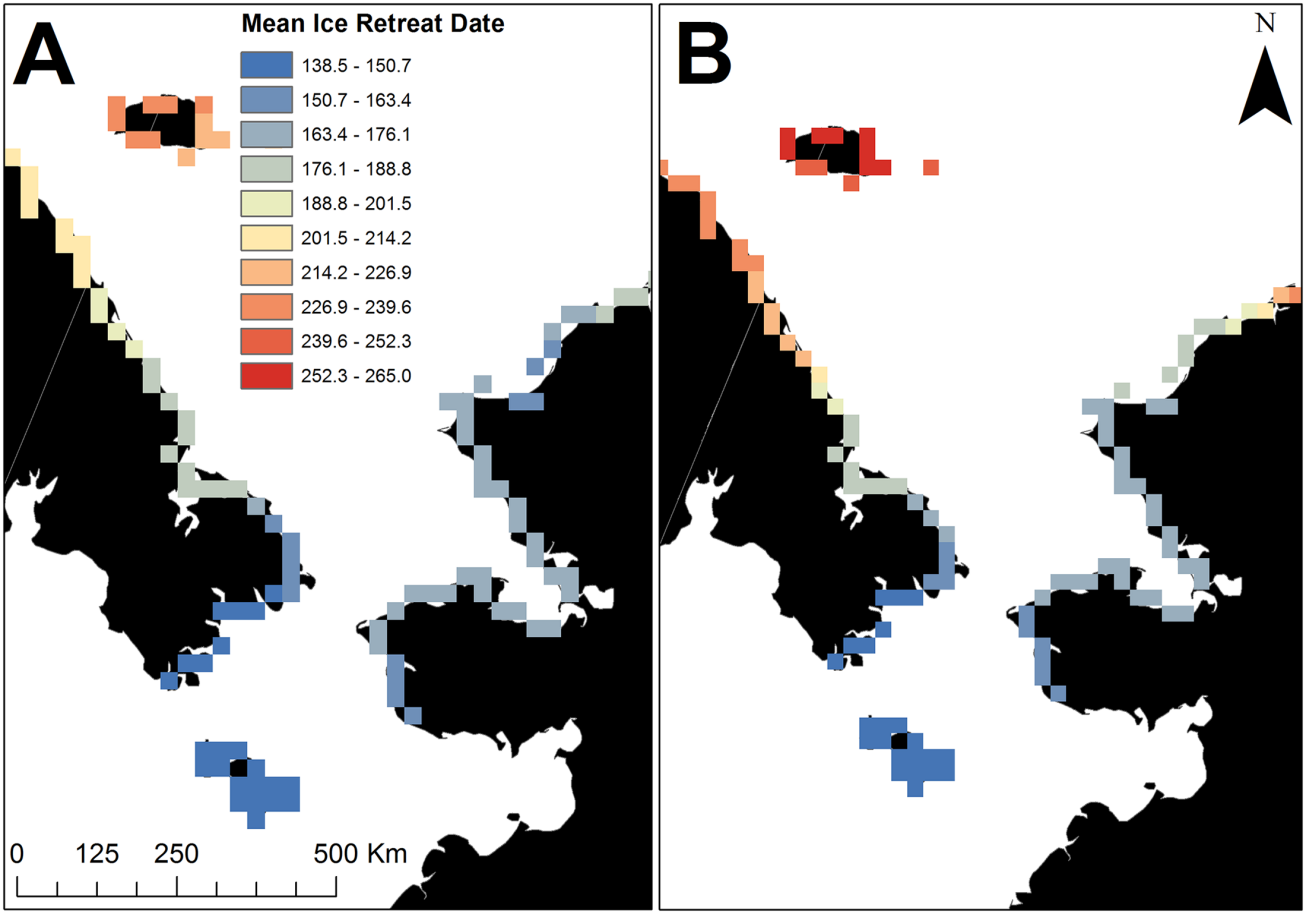
Mean ice retreat dates for 2008–2013 (A) and 1986–1995 (B). Hotter colors (reds) indicate that ice leaves the coast later during the retreat period and bears have access to the sea ice longer whereas cooler colors indicate that ice leaves the coast earlier.

**Fig 5:**
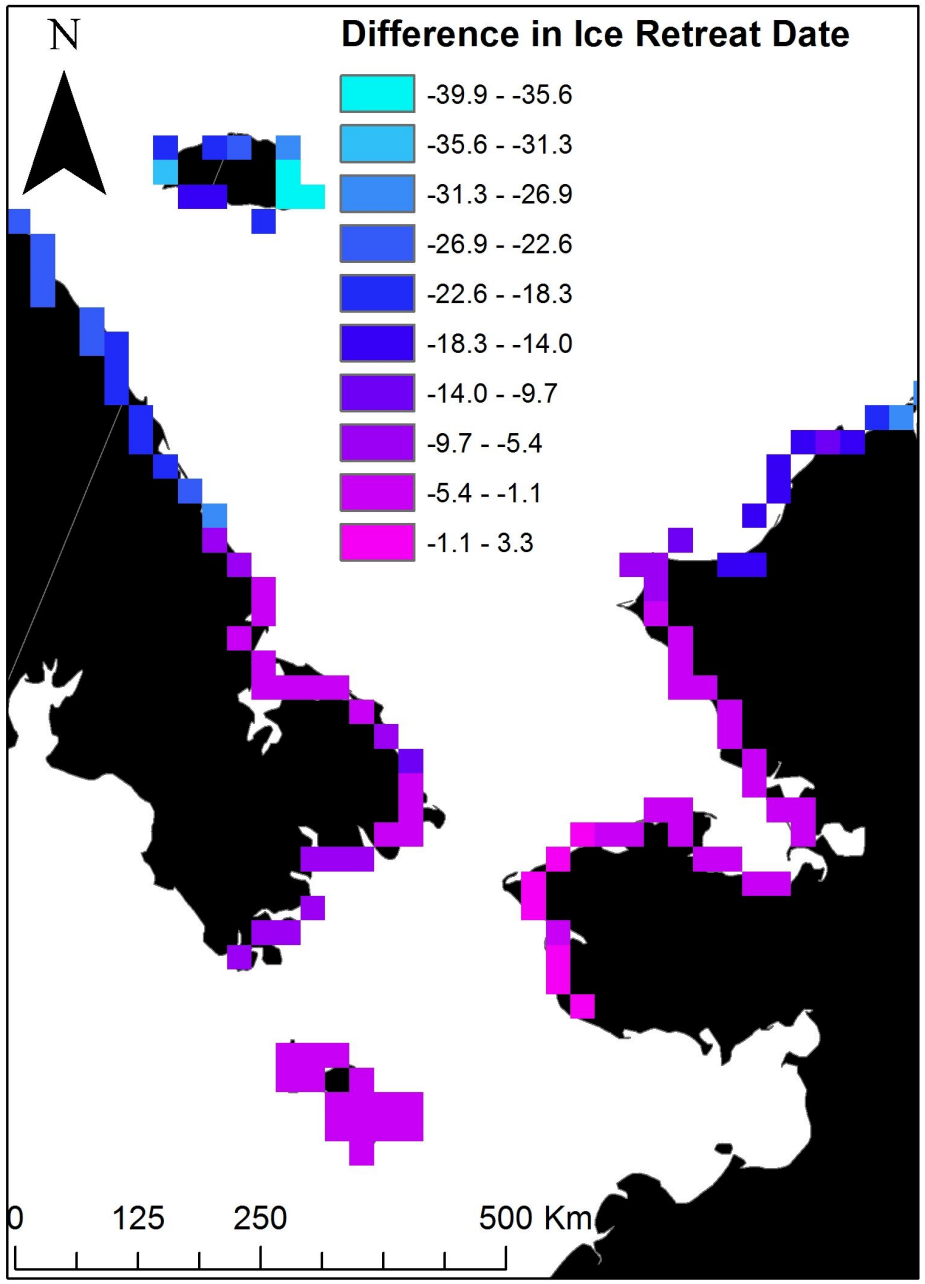
Differences in the ice retreat date along the Chukchi Sea coastline in Alaska and Russia between current (2008–2013) and historic (1986–1995) periods. Negative values indicate earlier ice retreat dates currently than during historic conditions. Units are in days.

We found no relationships between capture location and subsequent summering ($\chi_{4}^{2}$ = 5.8; *P* = 0.22) or den use locations ($\chi_{6}^{2}$ = 8.3; *P* = 0.22). The land mass and locations used during the summer and for denning by female polar bears were related to the timing of spring sea ice retreat. Across the Chukchi Sea subpopulation’s range, the ice retreat date was approximately 126 days later in northern parts of the study area compared to more southerly coastlines (Fig 4a and 4b). However, ice retreat dates were 20–40 days earlier in more northerly areas in 2008–2013 compared to 1986–1995 whereas they were 0–15 days earlier in more southerly areas (Fig 5). Although the locations where bears used land for both summering and for denning was related to ice return date, ice retreat date was the stronger predictor of the two metrics (S1 Table); thus we have presented only ice retreat results.

**Fig 6:**
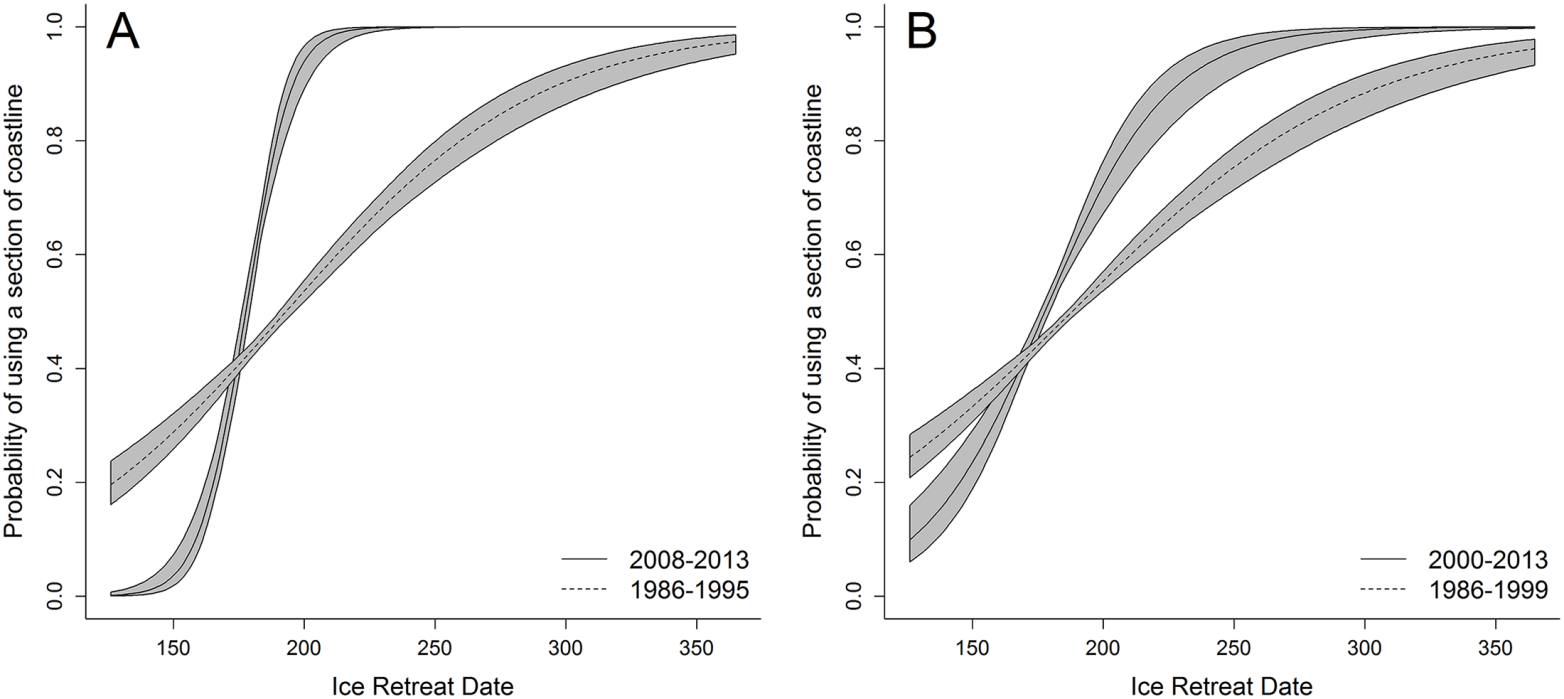
Relationship between the probability (± SE represented by the gray polygon) of a section of terrestrial, coastal habitat being used during the summer (i.e., for > 7 days between August and October) by female polar bears in 1986–1995 and 2008–2013 (A) or for denning in 1986–1999 and 2000–2013 (B) in the Chukchi Sea and ice retreat date (i.e., the first ordinal date in which ice of ≥15% concentration exceeded 50 km from the coastal location). Summering data were only available during the time periods included in (A) whereas some dens were identified between 1996–2007 (see Fig 3). Coastal habitats included locations in Alaska, Chukotka, and on Wrangel and Herald Islands.

The ice retreat date was highly predictive of whether a section of coastline (the 25km rasterized coastal segments as described in the methods) was used in the summer (*β* = 0.022, SE = 0.004, *P* < 0.001; Fig 6a) or for denning (*β* = 0.019, SE = 0.004, *P* < 0.001; Fig 6b), with bears selecting sections of coastline with later dates of ice retreat (S1 Table). This relationship changed over time for bears that spent time onshore during the summer, with bears in 2008–2013 showing significantly stronger selection for sections of coastline having later ice retreat dates (ice retreat date * time period interaction: *β* = 0.113, SE = 0.035, *P* = 0.001; Fig 6a). During both time periods, however, bears still exhibited positive selection for sections of coastline with later ice retreat dates. Similarly, bears selecting denning habitat differed in their selection of coastlines between periods (ice retreat date * time period interaction: *β* = 0.025, SE = 0.021, *P* = 0.03; Fig 6b).

## Discussion

Polar bears in the Chukchi Sea relied heavily on sea ice habitats with greater than 45% of adult females in the subpopulation spending 95% or more of their time on the sea ice (*n* = 110). Land, however, was also necessary for critical life history functions, with >84% of denning of this subpopulation occurring on land (*n* = 70). Our observation that there was no change in the proportion of bears denning on land between the two time periods suggests that, despite differences in capture location (e.g., that a higher proportion of bears were captured on Wrangel Island in the earlier period), land-based denning was and continues to be important to the Chukchi Sea subpopulation. This result was consistent when comparing denning location of bears captured in similar areas for both time periods. Furthermore, land appears to be becoming an increasingly important summer habitat for bears as evidenced by a doubling in the proportion of bears coming to shore for an extended period, and an increase of 30 days in the amount of time spent onshore. The earlier arrival of some bears onshore is consistent with observational studies conducted on Wrangel Island [14, 57]. The relationship between land tenure and sea ice conditions suggests that increased land use is likely a result of a longer period of reduced ice conditions. Ice retreat dates in the northern part of the study area were found to occur 20 to 40 days earlier during 2008–2013 compared to 1986–1995. Other observational studies have similarly noted the relationship between the timing of bears arrival and departure on land and sea ice conditions [57, 58].

Pregnant females commonly came onshore during the summer prior to denning in both the earlier (37.5%; n = 16) and latter period (57.1%; n = 14). This differs from the southern Beaufort Sea where bears that came onshore to feed on whale remains from subsistence harvests during the summer did not subsequently den on land [30]. Thus, summering and denning on land may be associated in the Chukchi subpopulation but is less so in the adjacent southern Beaufort Sea subpopulation. These subpopulations also differ in that a much greater proportion of the Chukchi Sea subpopulation has historically used land for denning, whereas in the southern Beaufort Sea, land-based denning increased from 38 to 63% between 1985–1995 and 1998–2004 in apparent response to reduced sea ice availability or quality [30]. Instead of increasingly using land for denning with reduced sea ice, pregnant females in the Chukchi Sea appear to be selecting for different land masses. We found that denning on the Alaskan coast declined by 15% while denning on Wrangel and Herald Islands and the Chukotkan coast increased by 15%. This result is consistent with traditional and local ecological knowledge collected among Alaska Native hunters who reported that in the past 10 years dens have only been observed at the village of Point Lay and to the north, whereas historically some denning was observed south of Point Lay ([59]; this study).

Our observation that ice was absent from southerly coastlines 126 days longer than more northerly coastlines suggests that denning in more southerly areas results in longer periods away from foraging habitats; and that perhaps a threshold in the southerly areas has been crossed that has decreased its use as a denning habitat. In land masses currently unused or minimally used for denning (e.g., the southern Alaska coast and far eastern Chukotkan coast) ice retreat begins approximately June 24 compared to more northern regions where ice retreat begins approximately Aug 6. Because sea ice loss in the Chukchi Sea is projected to continue [60], more northern land habitats, particularly Wrangel and Herald islands, may increasingly be used for summering and denning. Wrangel and Herald Islands currently support 68% of denning Chukchi Sea polar bears (this study; total proportion of all dens), and although Wrangel Island is designated as a Nature Reserve, it is currently being developed with both tourism and military infrastructure on some parts of the island. The viability of these habitats for denning may depend on human activities and continuation of the ice retreat date occurring after June 24. Our study is not the first to document changes in the distribution of den sites on land due to changes in the timing of sea ice melt and freeze [31]. Derocher et al. [31] found that in years with late ice advancement in autumn, polar bears in Svalbard, Norway, were much less likely to make use of a specific island to den than when ice was present earlier in autumn.

Polar bears have been regularly observed onshore in Chukotka in recent years despite none of the bears in our study spending more than 7 days onshore there between August and October. This could indicate that bears are using the Chukotka coast in the summer less than they have in the past because ice retreat occurs earlier there (15% of bears captured 1986–1995 summered in Chukotka). Nineteen percent of our sample of bears that denned on land denned on Chukotka. We did observe in our study that some bears summered on one land mass and moved to a different land mass for denning, including one bear that came onshore in Alaska, and one that came onshore on Wrangel Island, and both then later denned on the Chukotkan coast.

The nutritional implications of increased land use by polar bears in the Chukchi Sea depend on the energetic balance they achieve throughout the year. Given that polar bears primarily consume energetically-rich marine prey on the sea ice, long-term declines in sea ice availability have been associated with negative energetic and eventually demographic implications [24, 61, 62], although such relationships can be expected to exhibit spatial, temporal, and ecological complexity [34]. Increased durations on land can only be accommodated if bears come onshore in sufficient body condition to withstand longer periods of food deprivation, or obtain increased access to food while on shore. Although bears can reduce energetic requirements by reducing activity on land, they cannot reduce metabolic energy costs [28, 29]. On Wrangel Island, polar bears are reported to spend the majority of their time resting or walking [57]. But polar bears also congregate at walrus haulouts that occur on Wrangel Island and on the Chukotkan coast in some years, where they have been documented to hunt and kill live walruses as well as trigger stampedes and scavenge the resulting carcasses. These walrus haulouts occur every year in some locations on the Chukotkan coast, but in other areas, such as Wrangel Island, do not occur every year [58]. Polar bears in this region have also been observed opportunistically feeding on marine and terrestrial mammal carcasses, such as grey whales (*Eschrichtius robustus*), muskox (*Ovibos moschatus*) and reindeer (*Rangifer tarandus*)[58]. During peak lemming (*Synaptomys spp*.) years, polar bears actively dig them out of burrows as do grizzly bears [58]. Although land-based foraging on marine mammals and terrestrial plants and animals has been documented for Chukchi Sea polar bears, the contribution of terrestrial foods to energetic requirements during the onshore period is unknown. The lack of a change in the body condition and reproduction of Chukchi Sea polar bears during the time period of this study [34, 59] suggest that Chukchi Sea polar bears either come onshore with sufficient body fat or they are finding sufficient food resources on land (marine or terrestrial) to offset increased durations on land. The duration in which Chukchi Sea polar bears can remain on land before a threshold is reached and nutritional consequences occur is unknown.

Timing of departure of polar bears from land post-denning in this study (April 7 in 1986–1995 and March 27 in 2008–2013) was consistent with previous studies which reported den emergence occurring between March 20^th^ to April 5^th^ with bears spending an additional 0.5 to 7 days at the den site [63]. In recent years, 3 bears departed from dens prior to March 1 which could suggest that some bears may be compensating for coming to land earlier by departing early; however, whether these maternity denning attempts were successful is unknown. Bears could continue to successfully produce cubs by shifting the denning period earlier, or conversely they could be abandoning dens early if energetic reserves run out. Bears that emerge early from dens could face low food availability and colder temperatures.

The increased frequency and duration of land use we observed in this study and its association with reduced summer sea ice suggest that Chukchi Sea polar bears may continue to increase the amount of time they spend on land in the future. This pattern of increased land use in response to sea ice loss is consistent with other studies [5, 7, 9] and predictions for polar bears [17, 32] suggesting that one of the primary ways in which polar bears will respond to continued sea ice loss will be via increased use of Arctic coastal terrestrial habitats. This has implications for both human safety and polar bear conservation and, in light of increased human activity in the Arctic, will likely require proactive management to maintain terrestrial habitats for polar bears and mitigate human-polar bear interactions [17].

## Supporting Information

S1 Table Akaike Information Criteria (AIC) scores from conditional logistic regression examining the effects of time period (1986–1995 versus 2008–2013), and ice retreat or return date on the location of summering and denning polar bears in the Chukchi Sea. (DOCX)

## References

[1] Stroeve JC, Kattsov V, Barrett A, Serreze M, Pavlova T, Holland M, et al (2012) Trends in Arctic sea ice extent from CMIP5, CMIP3, and observations. Geophys Res Lett 39:28.

[2] Wassmann P, Duarter CM, Agusti S, Sejr MK (2011) Footprints of climate change in the Arctic marine ecosystem. Glob Change Biol 17:1235–1249.

[3] Obbard ME, Thiemann GW, Peacock E, DeBruyn TD (2010) Polar bears: Proceedings of the 15^th^ working meeting of the IUCN/SSC Polar Bear Specialist Group, Copenhagen, Denmark, 29 June– 3 July 2009. Gland, Switzerland and Cambridge, UK: IUCN Vii + 235 pp.

[4] Atkinson SN, Nelson RA, Ramsay MA (1996) Changes in the body composition of fasting polar bears (*Ursus maritimus*): the effect of relative fatness on protein conservation. Phys Zool 69:304–316.

[5] Schliebe S, Rode KD, Gleason JS, Wilder J, Proffitt K, Evans TJ, et al (2008) Effects of sea ice extent and food availability on spatial and temporal distribution of polar bears during the fall open-water period in the Southern Beaufort Sea. Polar Biol 31:999–1010.

[6] Aars J, Marques TA, Buckland ST, Andersen M, Belikov S, Boltunov A, et al (2009) Estimating the Barents Sea polar bear subpopulation size. Mar Mam Sci 25:35–52.

[7] Stirling I, Lunn NJ, Iacozza J (1999) Long-term trends in the population ecology of polar bears in western Hudson Bay in relation to climate change. Arctic 52:294–306.

[8] Cherry SG, Derocher AE, Thiemann GW, Lunn NJ (2013) Migration phenology and seasonal fidelity of an Arctic marine predator in relation to sea ice dynamics. J Anim Ecol 82:912–921. 10.1111/1365-2656.12050 23510081

[9] Prop J, Aars J, Bardsen B-J, Hanssen SA, Bech C, Bourgeon S, et al (2015) Climate change and the increasing impact of polar bears on bird populations. Frontiers in Ecology and Evolution: in press.

[10] Stempniewicz L (2006) Polar bear predatory behaviour toward molting Barnacle Geese and nesting glaucous gulls on Spitsbergen Arctic 59:247–251.

[11] Smith PA, Elliott KH, Gaston AJ, Gilchrist HG (2010) Has early ice clearance increased predation on breeding birds by polar bears? Polar Biol 33:1149–1153.

[12] Rockwell RF, Gormenzano LJ, Koons DN (2011) Trophic matches and mismatches: can polar bears reduce the abundance of nesting snow geese in western Hudson Bay Oikos 120:696–709.

[13] Iverson SA, Gilchrist HG, Smith PA, Gaston AJ, Forbes MR (2014) Longer ice-free seasons increase the risk of nest depredation by polar bears for colonial breeding birds in the Canadian Arctic. Proc Royal Soc B 281: 1779. 10.1098/rspb.2013.3128 PMC3924086 24500172

[14] Kochnev AA (2002) Autumn aggregations of polar bears on the Wrangel Island and their importance for the population. Proceedings of the Marine Mammals of the Holarctic meeting, Sept 10–15, 2002, Baikal, Russia.

[15] Miller S, Wilder J, Wilson RR (2015) Polar bear-grizzly bear interactions during the autumn open water period in Alaska. J Mamm: in press.

[16] Kelly B, Whitely A, Tallmon D (2010) The Arctic melting pot. Nature 468:891 10.1038/468891a 21164461

[17] Derocher AE, Aars J, Amstrup SC, Cutting A, Lunn NJ, Molnar PK, et al (2013) Rapid ecosystem change and polar bear conservation. Cons Let 6:368–375.

[18] Derocher AE (2012) Polar bears: a complete guide to their biology and behavior. John Hopkins Univ Press, Baltimore, MD, USA.

[19] Rode KD, Reist JD, Peacock E, Stirling I (2010) Comments in response to “Estimating the energetic contribution of polar bear (*Ursus maritimus*) summer diets to the total energy budget” by Dyck and Kebreab (2009). J Mamm 91:1517–1523.

[20] Rode KD, Robbins CT, Nelson L, Amstrup SC (2015) Can polar bears use terrestrial foods to offset lost ice-based hunting opportunities? Frontiers Ecol Env 13:138–145.

[21] Ramsay MA, Nelson RA, Stirling I (1991) Seasonal changes in the ratio of serum urea to serum creatinine in feeding and fasting polar bears. Can J Zool 69:298–302.

[22] Ramsay MA, Hobson K (1991) Polar bears make little use of terrestrial food webs: evidence from stable-carbon isotope analysis. Oecol 86:598–600. 10.1007/BF00318328 28313343

[23] Hobson KA, Stirling I, Andriashek DS (2009) Isotopic homogeneity of breath CO_2_ from fasting and berry-easting polar bears: implications for tracing reliance on terrestrial foods in a changing Arctic. Can J Zool 87:50–55.

[24] Regehr EV, Lunn NJ, Amstrup SC, Stirling I (2007) Effects of earlier ice breakup on survival and population size of polar bears in western Hudson Bay. J Wildl Manage 71:2673–2683.

[25] Lunn NJ, Servanty S, Regehr EV, Converse SJ, Richardson E, Stirling I (2014) Demography and population status of polar bears in western Hudson Bay Environment Canada Research Report, Edmonton, Canada.

[26] Amstrup SC, Marcot BG, Douglas DC (2008) A Bayesian network modeling approach to forecasting the 21^st^ century worldwide status of polar bears In: Arctic Sea Ice Decline: Observations, Projections, Mechanisms, and Implications (eds. DeWeaver E.T., Bitz C.M., and Tremblay L.B.). Pp 213–268 Geophys Monogr 180: American Geophysical Union, Washington, DC.

[27] Bengston JL, Hiruki-Raring LM, Simpkins MA, Boveng PL (2005) Ringed and bearded seal densities in the eastern Chukchi Sea, 1999–2000. Polar Biol 28:833–845.

[28] Whiteman JP, Harlow HJ, Durner GM, Anderson-Sprecher R, Albeke SE, Regehr EV et al (2015) Summer declines in activity and body temperature offer polar bears limited energy savings. Science 349:295–298. 10.1126/science.aaa8623 26185248

[29] Fischbach AS, Amstrup SC, Douglas DC (2007) Landward and eastward shift of Alaskan polar bear denning associated with recent sea ice changes. Polar Biol 30:1395–1405.

[30] Derocher AE, Andersen M, Wiig Ø, Aars J, Hansen E, and Biuw M (2011) Sea ice and polar bear den ecology at Hopen Island, Svalbard. Mar Ecol Progr Series 441:273–279.

[31] Derocher AE, Lunn LJ, Stirling I (2004) Polar bears in a warming climate. Integr Comp Biol 44:163–176. 10.1093/icb/44.2.163 21680496

[32] Freitas C, Kovas KM, Andersen M, Aars J, Sandven S, Skern-Mauritzen M, et al (2012) Importance of fast ice and glacier fronts for female polar bears and their cubs during spring in Svalbard, Norway. Mar Ecol Progr Series 447:289–304.

[33] Robbins CT, Lopez-Alfaro C, Rode KD, Tien O, Lynne Nelson O (2012) Hibernation and seasonal fasting in bears: the energetic costs and consequences for polar bears. J Mamm 93:1493–1503.

[34] Rode KD, Regehr EV, Douglas DC, Durner G, Derocher AE, Thiemann GW, et al (2014) Variation in the response of an Arctic top predator experiencing habitat loss: feeding and reproductive ecology of two polar bear populations. Glob Change Biol 20:76–88. 10.1111/gcb.12339 23913506

[35] Ovsyanikov NG, Menyushina IE (2008) Specifics of polar bear surviving ice free season on Wrangel Island in 2007 Proceedings of the Marine Mammals of the Holarctic. Odessa, Ukraine pp. 407–412.

[36] Uspenski SM, Kistchinski AA (1972) New data on the winter ecology of the polar bear (*Ursus maritimus* Phipps) on Wrangel Island. Int Con Bear Res Manage 2:181–198.

[37] Kochnev AA, Etylin VM, Kavry VI, Siv-Siv EB, Tanko IV (2003) Traditional knowledge of Chukotka native peoples regarding polar bear habitat use. Pp 1–166.

[38] Stirling I, Spencer C, Andriashek D (1989) Immobilization of polar bears (*Ursus maritimus*) with Telazol^®^ in the Canadian Arctic. J Wildl Dis 25: 159–168. 2716095 10.7589/0090-3558-25.2.159

[39] Douglas DC, Weinzierl R, Davidson SC, Kays R, Wikelski M, Bohrer G (2012) Moderating Argos location errors in animal tracking data. Meth Ecol Evol 3:999–1007.

[40] Johnson DS, London JM, Lea MA, Durban JW (2008) Continuous-time correlated random walk model for animal telemetry data. Ecol 89:1208–1215. 10.1890/07-1032.1 18543615

[41] Frair JL, Fieberg J, Hebblewhite M, Cagnacci F, DeCeasre NJ, Pedrotti L (2010) Resolving issues of imprecise and habitat-biased locations in ecological analyses using GPS telemetry data. Philosophical Transactions of the Royal Society B 365:2187–2200. 10.1098/rstb.2010.0084 PMC2894963 20566496

[42] R Development Core Team (2013) R: A language and environment for statistical computing. R Foundation for statistical computing, Vienna, Austria.

[43] Johnson DS (2013) Crawl: fit continuous-time correlated random walk models to animal movement data. R package version 1.4–1.

[44] Hooten MB, Hanks EM, Johnson DS, Alldredge MW (2014) Temporal variation and scale in movement-based resource selection functions. Statistical Methodology 17:82–98.

[45] Mahoney A.R., Eicken H., Shapiro L.H., Gens R., Heinrichs T., Meyer F.J., et al 2012 Mapping and characterization of recurring spring leads and landfast ice in the Beaufort and Chukchi Seas U.S. Department of the Interior. Bureau of Ocean Energy Management, Alaska Region, Anchorage, AK OCS study BOEM 2012–067. 179pp.

[46] Olson J, Rode KD, Smith T, Eggett D. 2015. Identifying maternal denning of polar bears using temperature: denning distribution in relation to sea ice in the southern Beaufort Sea. M.S. Thesis. Brigham Young University, Provo, Utah.

[47] Ferguson SH, Taylor MK, Rosing-Asvid A, Born EW, Messier F (2000) Relationships between denning of polar bears and conditions of sea ice. J Mam 81:1118–1127.

[48] Cavalieri DJ, Parkinson CL, Gloersen P, Zwally H (1996) Sea ice concentrations from Nimbus-7 SMMR and DMSP SSM/I-SSMIS passive microwave data Boulder, Colorado, USA NASA National Snow and Ice Data Center Distributed Active Archive Center.

[49] Durner GM, Douglas DC, Nielson RM, Amstrup SC (2006) A model for autumn pelagic distribution of adult female polar bears in the Chukchi Sea, 1987–1994. Anchorage, USGS Alaska Science Center, Contract Completion Report 70181-5-N240, 67 pp.

[50] Durner GM, Douglas DC, Nielson RM, Amstrup SC, McDonald TL, Stirling I, et al (2009) Predicting 21^st^-century polar bear habitat distribution from global climate models. Ecol Monogr 79:25–58.

[51] VanDerWal J, Falconi L, Januchowski S, Shoo L, Storlie C (2014) SDMTools: species distribution modelling tools: tools for processing data associated with species distribution modelling exercises. R package version 1.1–221.

[52] Pagano AM, Durner GM, Amstrup SC, Simac KS, York GS (2012) Long-distance swimming by polar bears (*Ursus maritimus*) of the southern Beaufort Sea during years of extensive open water. Can J Zool 90:663–676.

[53] Manly BFJ, McDonald LL, Thomas DL, McDonald TL, Erickson WP (2002) Resource selection by animals. Kluwer Academic Publishers, Dordrecht, The Netherlands.

[54] Therneau T (2013) A package for survival analysis in S. R package version 2.37‒4.

[55] Burnham KP, Anderson DR (2002) Model Selection and Multimodel Inference: a Practical Information-theoretic Approach. 2nd Edn Springer, NY.

[56] Zar JH (1999) Biostatistical analysis. Pearson Education India.

[57] Ovsyanikov NG, Menyushina IE (2010) Number, condition, and activity of polar bears on Wrangel Island during ice free autumn seasons of 2005–2009. Proceedings of the Marine Mammals of the Holarctric meeting. Oct 11–15, 2010. Kalinigrad, Russia.

[58] Ovsyanikov NG (2005) Polar bear behavior in coastal congregations. Zool J 84:94–103.

[59] Voorhees H, Sparks R, Huntington HP, Rode KD (2014) Traditional knowledge of polar bears (*Ursus maritimus*) in Northwestern Alaska. Arctic 67: in press.

[60] Stroeve JC, Markus T, Boisvert L, Miller J, Barrett A (2014) Changes in Arctic melt season and implications for sea ice loss. Geophys Res Let 41:1216–1225.

[61] Regehr EV, Hunter CM, Caswell H, Amstrup SC, Stirling I (2009) Survival and breeding of polar bears in the southern Beaufort Sea in relation to sea ice. J Anim Ecol 79:117–127. 10.1111/j.1365-2656.2009.01603.x 19754681

[62] Rode KD, Amstrup SC, Regehr EV (2010) Reduced body size and cub recruitment in polar bears associated with sea ice decline. Ecol App 20:768–782. 10.1890/08-1036.1 20437962

[63] Uspenski SM, Kistchinski AA (1972) New data on the winter ecology of the polar bear (*Ursus maritimus* Phipps) on Wrangel Island Bears: their biology and management. Vol 2, pp. 181–197.

