# Supplementary material for: Increased Land Use by Chukchi Sea Polar Bears in Relation to Changing Sea Ice Conditions
Source: PLoS One. 2015 Nov 18;10(11):e0142213. doi: 10.1371/journal.pone.0142213 (PMC4651550; doi:10.1371/journal.pone.0142213)
Supplement: S1 Table — (DOCX) [file pone.0142213.s001.docx]

**S1 Table. Akaike Information Criteria (AIC) scores from conditional logistic regression examining the effects of time period (1986-1995 versus 2008-2013), and ice retreat or return date on the location of summering and denning polar bears in the Chukchi Sea.**

|  | AIC | |
| --- | --- | --- |
| Model | Ice Retreat Date | Ice Return Date |
| Summering | 305.8 | 326.4 |
| Denning | 490.8 | 500.5 |
